# Unifying cancer and normal RNA sequencing data from different sources

**DOI:** 10.1038/sdata.2018.61

**Published:** 2018-04-17

**Authors:** Qingguo Wang, Joshua Armenia, Chao Zhang, Alexander V. Penson, Ed Reznik, Liguo Zhang, Thais Minet, Angelica Ochoa, Benjamin E. Gross, Christine A. Iacobuzio-Donahue, Doron Betel, Barry S. Taylor, Jianjiong Gao, Nikolaus Schultz

**Affiliations:** 1 Human Oncology and Pathogenesis Program, Memorial Sloan Kettering Cancer Center, New York, New York 10065, USA; 2 Marie-Josée and Henry R. Kravis Center for Molecular Oncology, Memorial Sloan Kettering Cancer Center, New York, New York 10065, USA; 3 College of Computing & Technology, Lipscomb University, Nashville, Tennessee 37204, USA; 4 Institute for Computational Biomedicine, Weill Cornell Medicine, New York, New York, 10021, USA; 5 Department of Pathology, Memorial Sloan Kettering Cancer Center, New York, New York 10065, USA; 6 Department of Epidemiology and Biostatistics, Memorial Sloan Kettering Cancer Center, New York, New York 10065, USA

**Keywords:** Cancer genomics, RNA sequencing, Data processing

## Abstract

Driven by the recent advances of next generation sequencing (NGS) technologies and an urgent need to decode complex human diseases, a multitude of large-scale studies were conducted recently that have resulted in an unprecedented volume of whole transcriptome sequencing (RNA-seq) data, such as the Genotype Tissue Expression project (GTEx) and The Cancer Genome Atlas (TCGA). While these data offer new opportunities to identify the mechanisms underlying disease, the comparison of data from different sources remains challenging, due to differences in sample and data processing. Here, we developed a pipeline that processes and unifies RNA-seq data from different studies, which includes uniform realignment, gene expression quantification, and batch effect removal. We find that uniform alignment and quantification is not sufficient when combining RNA-seq data from different sources and that the removal of other batch effects is essential to facilitate data comparison. We have processed data from GTEx and TCGA and successfully corrected for study-specific biases, enabling comparative analysis between TCGA and GTEx. The normalized datasets are available for download on figshare.

## Background & Summary

RNA sequencing (RNA-seq) is an important tool for understanding the genetic mechanisms underlying human diseases. Large-scale sequencing studies have recently generated a great wealth of RNA-seq data. For example, The Cancer Genome Atlas (TCGA) has quantified gene expression levels in >8000 samples from >30 cancer types. On a similar scale, the Genotype Tissue Expression (GTEx) project^1,2^, has catalogued gene expression in >9,000 samples across 53 tissues from 544 healthy individuals.

These resources offer a unique opportunity to gain better insight into complex human diseases. However, the integrative analysis of these data across studies poses great challenges, due to differences in sample handling and processing, such as sequencing platform and chemistry, personnel, details in the analysis pipeline, etc. For example, the RNA-seq expression levels of the majority of genes quantified are in the range of 4-10 (log2 of normalized_count) for TCGA, and 0-4 (log2 of RPKM) for GTEx (Supplementary Fig. S1A), a consequence of the use of different analysis pipelines. This makes gene expression levels from the two projects not directly comparable.

To facilitate research on abnormal gene expression in human diseases, a variety of databases and pipelines have been developed to combine RNA-seq from different studies^3–10^. However, these databases or pipelines either directly incorporated expression data from the literature, retaining unwanted batch effects in the data^7,8^, or only combined and reanalyzed samples from smaller studies, hence, not taking advantage of the power provided by the recent large data sets^3–6,10^. A recently published pipeline, the Toil RNA-seq Pipeline^11^, attempts to unify RNA-seq data from different sources by uniformly processing raw sequencing reads. However, Toil does not remove batch effects that are introduced by sources other than the differences in read alignment and quantification. To take full advantage of the large volume of available RNA-seq data, an integrative RNA-seq resource is necessary.

Here, we developed a pipeline for processing and unifying RNA-seq data from different studies. By unifying data from GTEx and TCGA, we provide reference expression levels across the human body for comparison with the expression levels found in human cancer. Our method removes batch effects by uniformly reprocessing RNA-seq data. Specifically, we used raw sequencing reads of the RNA-seq samples downloaded from GTEx and TCGA, realigned them, re-quantified gene expression, and then removed biases specific to each study.

## Methods

### RNA-seq data

Raw paired-end reads of the RNA-seq samples for the TCGA project were retrieved from the Cancer Genomics Hub (CGHub, https://cghub.ucsc.edu). When FASTQ files were not available, e.g., for stomach adenocarcinoma, we downloaded aligned sequence reads (in BAM format) and extracted reads from BAM files with the Java program ubu.jar (https://github.com/mozack/ubu) before processing samples using our pipeline. GTEx samples were downloaded from the Database of Genotypes and Phenotypes (dbGaP, http://www.ncbi.nlm.nih.gov/gap), which hosts >9,000 RNA-seq samples (in SRA format) for the GTEx study.

### Analysis pipeline

**Figure 1:**
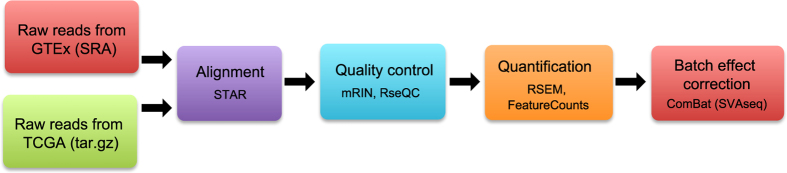
Uniform processing of RNA-seq data from GTEx and TCGA.

Our analysis pipeline included realignment of raw reads, removal of degraded samples, expression quantification, and batch effect processing (Fig. 1).

We employed STAR aligner^12^, a fast accurate alignment software used widely in the NGS community, to map reads to UCSC human reference genome hg19 and reference transcriptome GENCODE (v19), using recommended parameters, e.g., ‘—outFilterType BySJout’ and ‘—outFilterMultimapNmax 20’, etc., which are also standard options of the ENCODE project for long RNA-seq pipeline. Samples with alignment rates less than 40% were excluded from further analysis.

The software tools FastQC, Picard (http://picard.sourceforge.net/index.shtml), RseQC^13^, and mRIN^14^ were used to evaluate sample quality. RNA degradation, as detected by mRNA, was present in some GTEx and TCGA samples. Since degradation can bias expression level measurements and cause data misinterpretation, we decided to exclude samples with evidence for degradation. To determine an appropriate degradation cutoff for mRIN, we used prostate cancer samples from the TCGA project, which had undergone extensive pathological, analytical, and quality control review and which had been shown to include a significant portion of degraded samples^15^. We used -0.11 as the degradation threshold for mRIN: samples with mRIN<−0.11 were regarded as degraded and, thus, excluded from further analysis.

To verify mRIN’s performance on other tissues, we manually examined coverage uniformity over gene bodies for other tissues using the tool RseQC^13^ and compared it with mRIN scores. We calculated the number of reads covering each nucleotide position and the average coverage for all long genes (>4000 nt). Supplementary Fig. S3 shows the average coverage for TCGA prostate and bladder samples, each curve representing gene body coverage of a sample. In Supplementary Fig. S3A, the 4 samples with the most uneven coverage are the ones deemed degraded. We made similar observations in the other tissues examined, e.g., bladder in Supplementary Fig. S3B, where the samples with the most imbalanced gene body coverage were the ones with the lowest mRIN scores. These results confirmed that mRIN is capable of measuring degradation for other tissues.

When running STAR, we specified an option ‘—quantMode TranscriptomeSAM’ to make STAR output a file, Aligned.toTranscriptome.out.bam, which contains alignments translated into transcript coordinates. This file was then used with RSEM^16^ to quantify gene expression. The program ‘rsem-calculate-expression’ in the RSEM package requires strand specificity of the RNA-seq sample, which is estimated using RseQC^13^.

We also used the transcript quantification tool FeatureCounts^17^ to generate integer-based read counts. Overall, the output of FeatureCounts was highly consistent with that of RSEM (Spearman correlation > 0.95). However, for genes with multi-mapping reads (i.e., reads mapped to multiple genes), FeatureCounts differs from RSEM and tends to underestimate expression levels in comparison with RSEM (because it discards multi-mapping reads). For example, the transcript of the *PGA3* gene, which encodes the human pepsinogen A enzyme, which is highly abundant in the stomach, is identical to the transcripts of two other genes, *PGA4* and *PGA5*. Its measurement in stomach by FeatureCounts (in default settings) is generally lower than that by RSEM (see Supplementary Fig. S4). In the section Technical Validation below, we primarily used results by RSEM.

We ran ComBat in the R package SVAseq^18,19^ to correct for non-biological variation accounting for unwanted differences between GTEx and TCGA samples of a particular tissue type. To ensure that TCGA normal samples remain comparable with TCGA tumors after removing batch biases from the normal samples, we processed TCGA tumors in the same way as the normal samples using our pipeline from raw sequencing reads. Both TCGA tumors and normal samples were adjusted together by including them in the same sample-gene matrix. In Supplementary Table S2, we used bladder and lung as examples to show the parameters we used to run ComBat (parameters used for other tissues are provided in a configuration file at https://github.com/mskcc/RNAseqDB/blob/master/configuration/tissue-conf.txt). As indicated in Supplementary Table S2, we treated all TCGA samples, both tumors and normal samples (of the same tissue type), as one batch. ComBat requires the creation of a model matrix to indicate the variables to be adjusted and variables of interest. In our model matrix, as shown in Supplementary Table S2, batch is treated as an adjustable variable and tumor / normal indicator a variable of interest.

### Principal component analysis

To perform principal component analysis, we first remove genes with invariant expression levels and then log_2_-transformed the sample-gene matrix. Next, we utilized the R function ‘prcomp’ (with the ‘center’ option set to TRUE) to perform principal component analysis. The two-dimensional PCA plot was created using the R function ‘autoplot’.

### Hierarchical clustering

For hierarchical clustering of expression data, we used the R function Heatmap.3 using default parameters (e.g., distance: Euclidean, hierarchical clustering method: Ward, etc.) as well as the 1000 most variable genes in the data matrix.

### Code availability

The detailed parameters we used to run STAR, RSEM and other tools and the codes of our pipeline are available at GitHub (https://github.com/mskcc/RNAseqDB). The versions of the tools, e.g., STAR and RSEM, are described in a README file at https://github.com/mskcc/RNAseqDB/blob/master/README.md.

## Data Records

The data generated using our pipeline is available on figshare (Data Citation 1,Data Citation 2, and Data Citation 3).

### Data record 1

The maximum likelihood gene expression levels computed using RSEM, i.e., the expected_count in RSEM’s output, are in Data Citation 1. This dataset includes 52 data files, each being a sample-gene matrix of a certain tissue type (see Table 1 for the tissues we processed). This dataset can be provided to programs such as edgeR for identifying differentially expressed genes.

### Data record 2

The gene expression levels calculated from the FPKM (Fragments Per Kilobase of transcript per Million) in RSEM’s output are in Data Citation 2. This dataset (of data files) was quantile normalized, but not corrected for batch effects.

### Data record 3

The normalized gene expression levels (FPKM) are in Data Citation 3. This dataset was not only quantile normalized, but was corrected for batch effects (using ComBat).

## Technical Validation

To allow proper batch bias correction, we processed only samples from tissues that were studied by both GTEx and TCGA (Table 1). Tissues with no or insufficient numbers of normal samples available in TCGA (e.g., sarcoma, ovarian cancer, melanoma) were not processed (supplementary Table S1).

We downloaded and processed raw paired-end RNA-seq data from 10,366 samples, including 2,790 from GTEx and 7,576 from the TCGA project (Table 1). 831 samples (8%) exhibited 5′ degradation (as described previously^15^) and were excluded from further analysis. We also discarded samples with low alignment rates and samples not used in the final GTEx study, resulting in a total of 9109 (89%) high-quality samples for further analysis.

To correct for batch biases, we first created a sample-gene matrix for each tissue-tumor pair by merging gene expression levels of the corresponding GTEx and TCGA samples. Regardless of the actual batch that a sample belonged to in an RNA-seq experiment, we treated all GTEx samples as one batch and TCGA samples as another. Then, we ran ComBat^18^ to correct for non-biological variation accounting for unwanted differences between GTEx and TCGA samples of a particular tissue type (see Methods).

**Figure 2:**
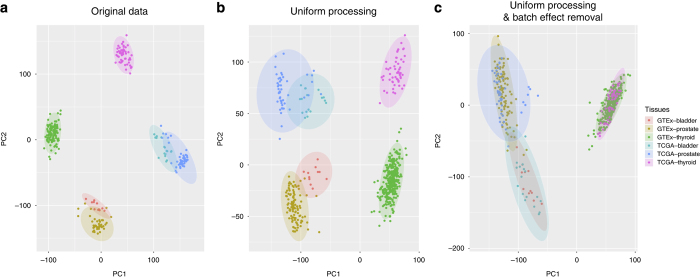
Effect of uniform processing and batch effect removal on gene expression levels in GTEx and TCGA. Two-dimensional plots are shown of principal components calculated by performing PCA of the gene expression values of bladder, prostate, and thyroid samples from GTEx and TCGA. **(a)** PCA of the level 3 data, i.e., the expression data from GTEx and TCGA. GTEx expression data was quantile normalized (see Supplementary Fig. S1B). **(b)** PCA of the expression data after uniform processing through our pipeline, before batch bias correction. **(c)** PCA of the expression data after uniform processing through our pipeline, after batch bias correction.

To examine how well our pipeline was able to correct study-specific batch effects, we systematically compared the effects of uniform realignment, expression quantification, and batch effect correction for three tissues: bladder, prostate and thyroid. When using expression levels reported by the TCGA and GTEx projects, even after applying upper-quartile normalization to bring expression levels into comparable ranges (Supplementary Fig. S1B), samples from the same study were more similar to each other than samples from the same tissue, as shown by PCA analysis (Fig. 2a). This result indicates the necessity to uniformly reprocess RNA-seq samples.

However, uniform realignment and expression quantification using our pipeline did not fully resolve these differences; while the first principal component was now the tissue, the second principal component was still defined by the source (Fig. 2b), indicating that study-specific biases still accounted for significant variation in RNA-seq expression levels within each tissue type. This result shows that consistent realignment and expression quantification alone are not sufficient, and that further study-specific batch effects need to be removed in order to be able to compare expression data from TCGA and GTEx.

To this end, we next added a batch-effect correction step to our pipeline, using ComBat^18^ (see Methods), which successfully corrected our example data and resulted in clustering by tissue type (Fig. 2c).

**Figure 3:**
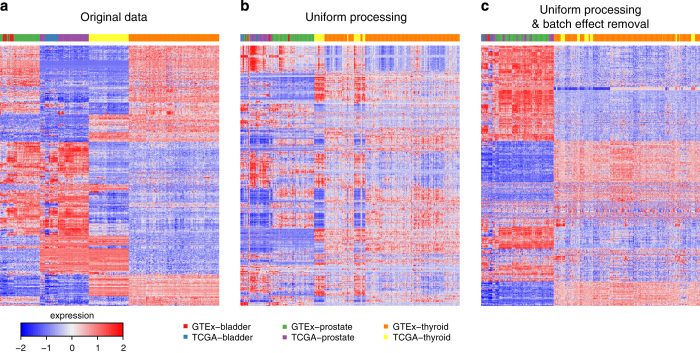
Hierarchical clustering of GTEx and TCGA bladder, prostate, and thyroid data shows the effect of uniform processing and batch effect correction. **(a)** level 3 expression data from GTEx and TCGA; **(b)** gene expression calculated using our pipeline prior to batch bias correction; **(c)** our expression data after batch bias correction.

To determine whether uniform alignment and expression quantification was an essential step, or whether batch effect removal via ComBat by itself was sufficient, we also applied ComBat directly to the level 3 data from GTEx and TCGA (GTEx-quantified data was rescaled using quantile normalization). We found that batch effect removal by itself is not sufficient, and that the combination of uniform processing of sequencing reads followed by additional batch effect removal is required to make data from the TCGA and GTEx projects comparable (Supplementary Fig. S2). We validated the expression similarities observed in the principal component analysis through hierarchical clustering (Fig. 3).

Our results demonstrate that uniform realignment and expression quantification, together with explicit correction for study-specific biases, are not only effective, but also necessary for removing batch effects and making samples from different studies comparable.

The bladder is more proximal (and developmentally closer) to prostate than to thyroid, and this tissue proximity is reflected in Fig. 2c. If three distal tissues, such as breast, lung, and liver, are used, the clusters representing the three biological subgroups will be more separated accordingly in the principle component analysis (Supplementary Fig. S5).

Here, we corrected batch biases for each tissue separately. We also evaluated a different strategy to remove batch biases between TCGA and GTEx as a whole. For the three tissue types processed through our pipeline, we used all TCGA normals as one batch and GTEx normals as another batch to run ComBat. Our preliminary analysis showed this strategy was not effective in making RNA-seq samples from the two studies comparable (Supplementary Fig. S6).

**Figure 4:**
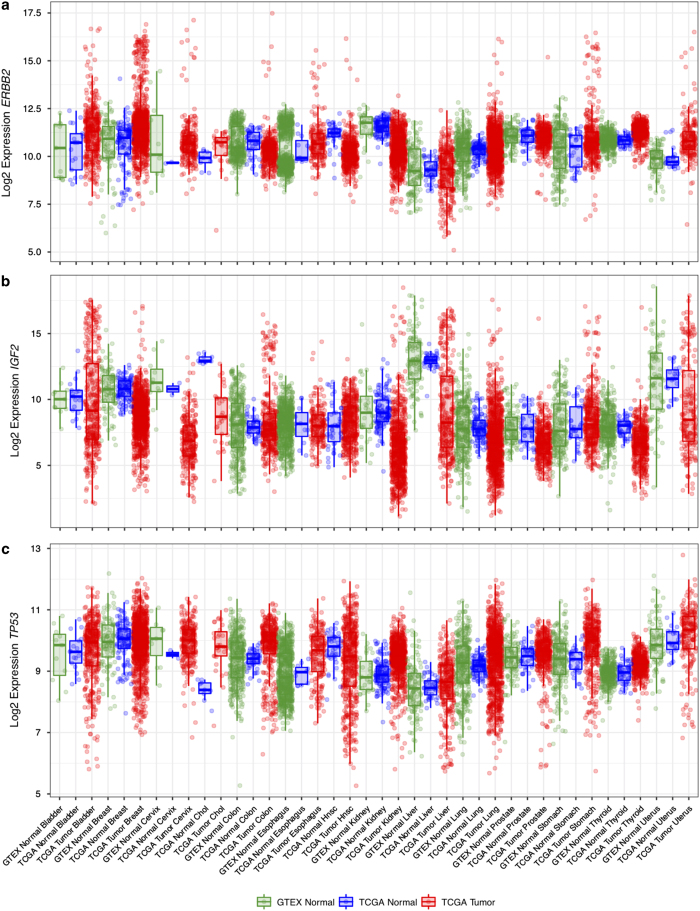
Normalized expression across tissue and cancer types for three known cancer genes: *ERBB2*, *IGF2* and *TP53*.

Finally, we examined the expression levels of three cancer driver genes, *ERBB2*, *IGF2*, and *TP53*, in our batch-effect corrected data (Fig. 4). *ERBB2* expression was significantly higher in a subset of tumor samples, consistent with the frequent amplifications observed in various tumor types. *IGF2* showed a similar pattern, with a subset of tumor samples expressing the gene at levels several orders of magnitude higher than those in normal samples. *TP53*, on the other hand, is often affected by truncating mutations in cancer, which leads to decreased levels of RNA due to nonsense-mediated decay, an effect that is visible in the normalized RNA data.

## Additional information

**How to cite this article:** Wang, Q. *et al.* Unifying cancer and normal RNA sequencing data from different sources. *Sci. Data* 5:180061 doi: 10.1038/sdata.2018.61 (2018).

**Publisher’s note:** Springer Nature remains neutral with regard to jurisdictional claims in published maps and institutional affiliations.

## Supplementary Material



Supplementary File

## Figures and Tables

**Table 1:** GTEx and TCGA RNA-seq samples processed by our pipeline.

**GTEx tissue / TCGA cancer type**	**GTEx**	**TCGA normal**	**TCGA tumor**	**Total**
bladder / blca	11	19	411	441
breast / brca	218	114	1112	1444
cervix / cesc	11	3	304	318
uterus / ucec	90	24	180	294
uterus / ucs		0	57	57
colon-sigmoid / read	173	10	94	277
colon-transverse / coad	203	41	295	539
liver / lihc	136	50	371	557
salivary gland / hnsc	70	44	520	634
esophageal / esca	790	11	185	986
prostate / prad	119	52	497	668
stomach / stad	204	35	415	654
thyroid / thca	355	59	505	919
lung / luad	374	59	528	961
lung / lusc		51	504	555
kidney cortex / kirc	36	72	541	649
kidney cortex / kirp		32	290	322
kidney cortex / kich		25	66	91
**Total**	2790	701	6875	10366
Only paired-end RNA-seq samples were included.				
