# Supplementary material for: Unifying cancer and normal RNA sequencing data from different sources
Source: Sci Data. 2018 Apr 17;5:180061. doi: 10.1038/sdata.2018.61 (PMC5903355; doi:10.1038/sdata.2018.61)
Supplement: Supplementary File [file sdata201861-s2.pdf]

Supplementary information for *Unifying cancer and normal RNA sequencing data from different sources*

Supplementary Figures	Page
**Figure S1**. **(a)** Ranges of GTEx and TCGA RNA-seq gene expression levels in bladder normal samples, as obtained from GTEx and TCGA, without any additional normalization. Expression values from both projects are on different scales and can therefore not be compared directly without further processing or normalization. **(b)** Gene expression levels in GTEx samples were scaled using quantile normalization.	1
**Figure S2.** PCA plot after applying quantile normalization and ComBat to the level 3 data of the 3 tissues, bladder, prostate, and thyroid, from GTEx and TCGA.	2
**Figure S3**. Gene body coverage of the TCGA prostate and bladder samples. Each curve in the figure represents average coverage of genes (from 5' to 3') in a sample – the different colors are used to indicate the different samples. To ease visual examination, only long genes (>4000 nt) were used in the calculation of the coverage and only the normal samples were plotted. Samples that were excluded due to a 3'/5' bias are shown with dashed lines.	3
**Figure S4**. Expression of the gene *PGA3* in six tissues. Gene expression in (a) and (b) were quantified using FeatureCounts and RSEM, respectively. The same set of GTEx and TCGA (both tumor and normal) samples was used to compare FeatureCounts and RSEM for each tissue type.	4
**Figure S5**. Two-dimensional plots of principal components calculated by performing PCA of the batch-corrected gene expression in breast, liver, and lung samples from GTEx and TCGA.	5
**Figure S6**. Effect of removing batch biases between TCGA and GTEx as a whole. For the three tissue types, bladder, prostate, and thyroid, processed through our pipeline, all TCGA normal samples were used as one batch and GTEx normal samples as another batch to run ComBat. Two-dimensional plots are shown of principal components calculated by performing PCA on the gene expression values after removing batch biases.	6
**Supplementary Tables**	
**Table S1.** Samples with no or insufficient numbers of normal samples available in TCGA or GTEx.	7
**Table S2.** Parameters of ComBat for: (a) bladder, and (b) lung. TCGA lung cancer has two subtypes: lung adenocarcinoma (LUAD) and lung squamous cell carcinoma (LUSC). LUSC was designated in the same batch as LUAD.	8

a. b.

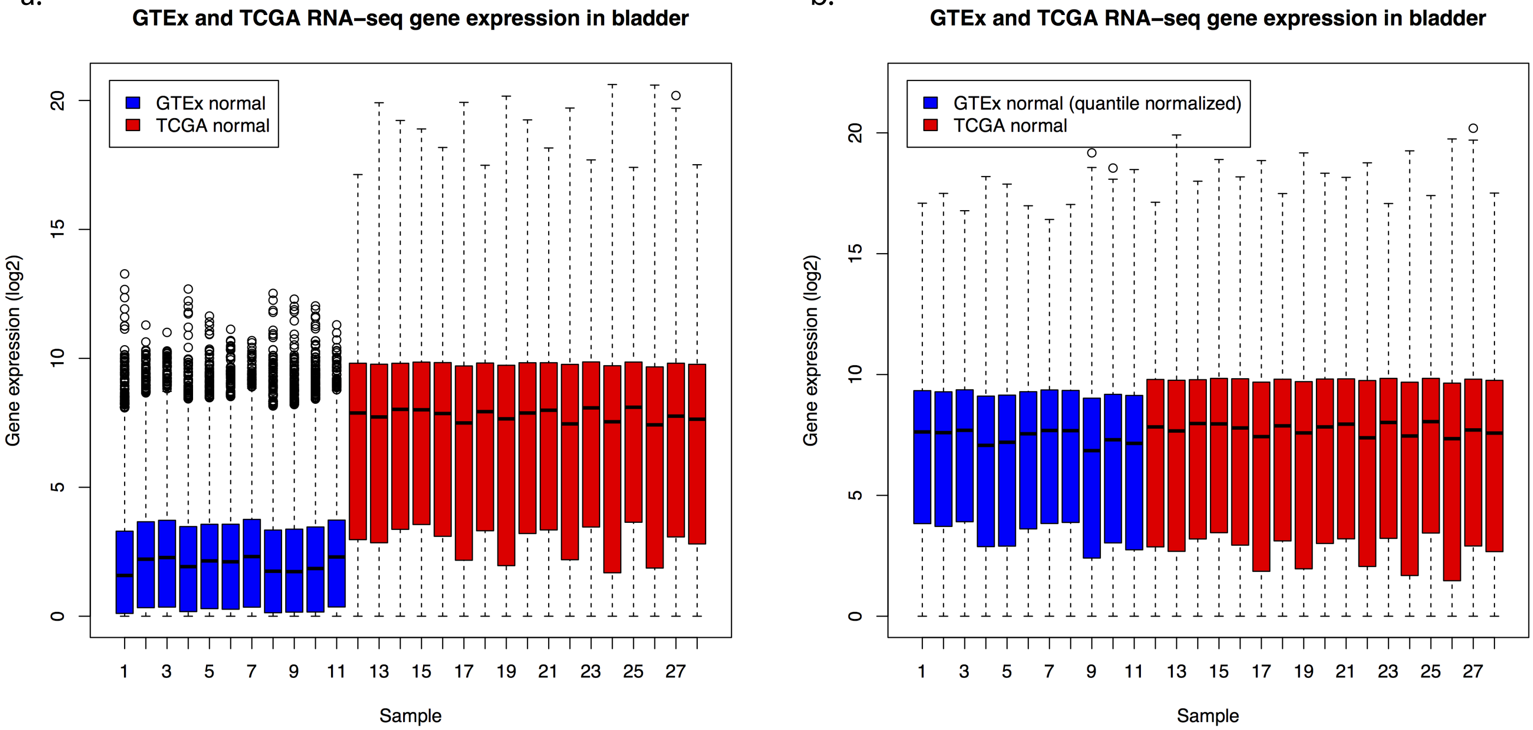
GTEx and TCGA RNA–seq gene expression in bladder
GTEx normal
TCGA normal
Gene expression (log2)
Sample
GTEx and TCGA RNA–seq gene expression in bladder
GTEx normal (quantile normalized)
TCGA normal
Gene expression (log2)
Sample

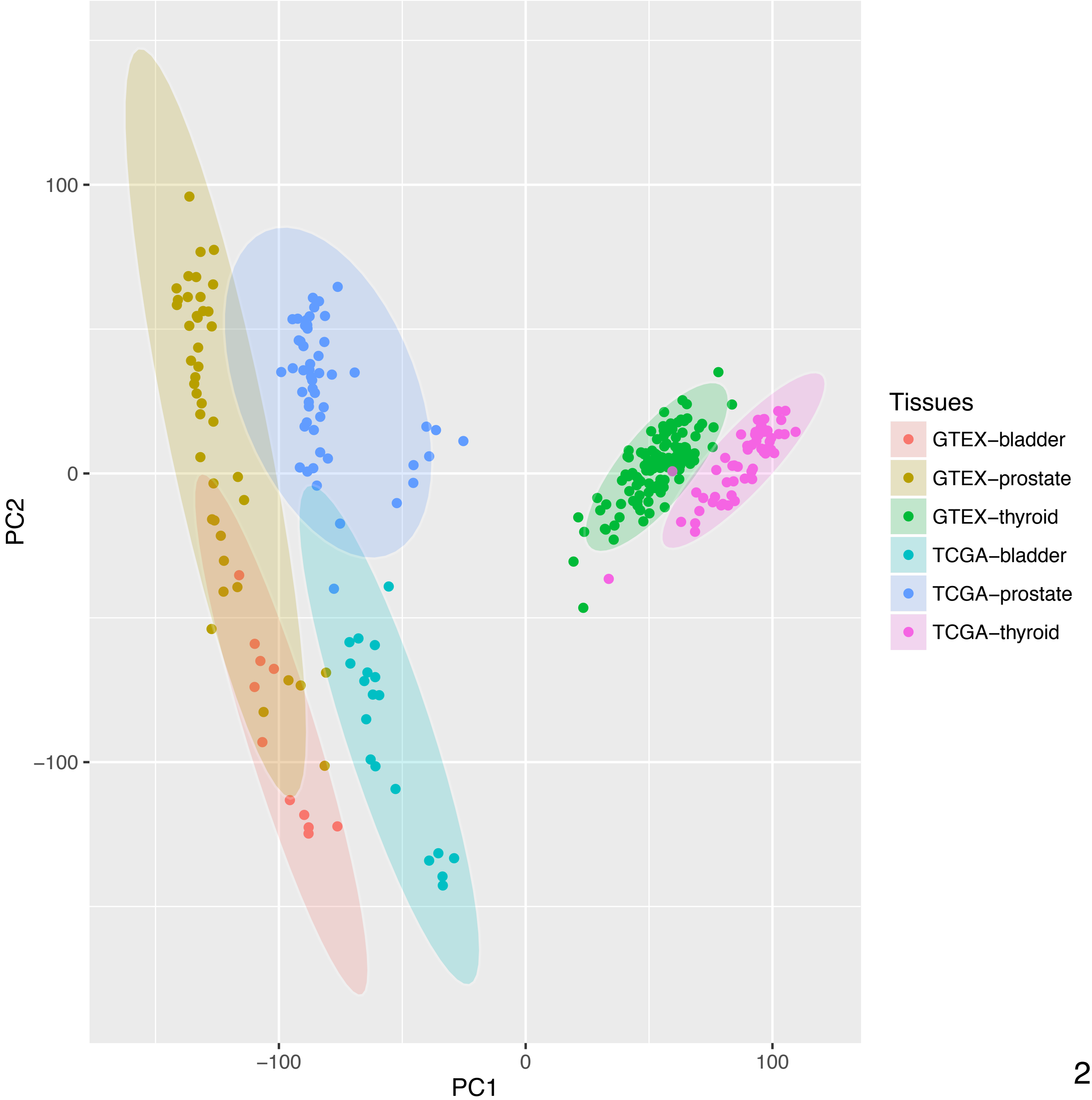

100
0
−100
PC2
−100
0
100
PC1
Tissues
GTEX−bladder
GTEX−prostate
GTEX−thyroid
TCGA−bladder
TCGA−prostate
TCGA−thyroid

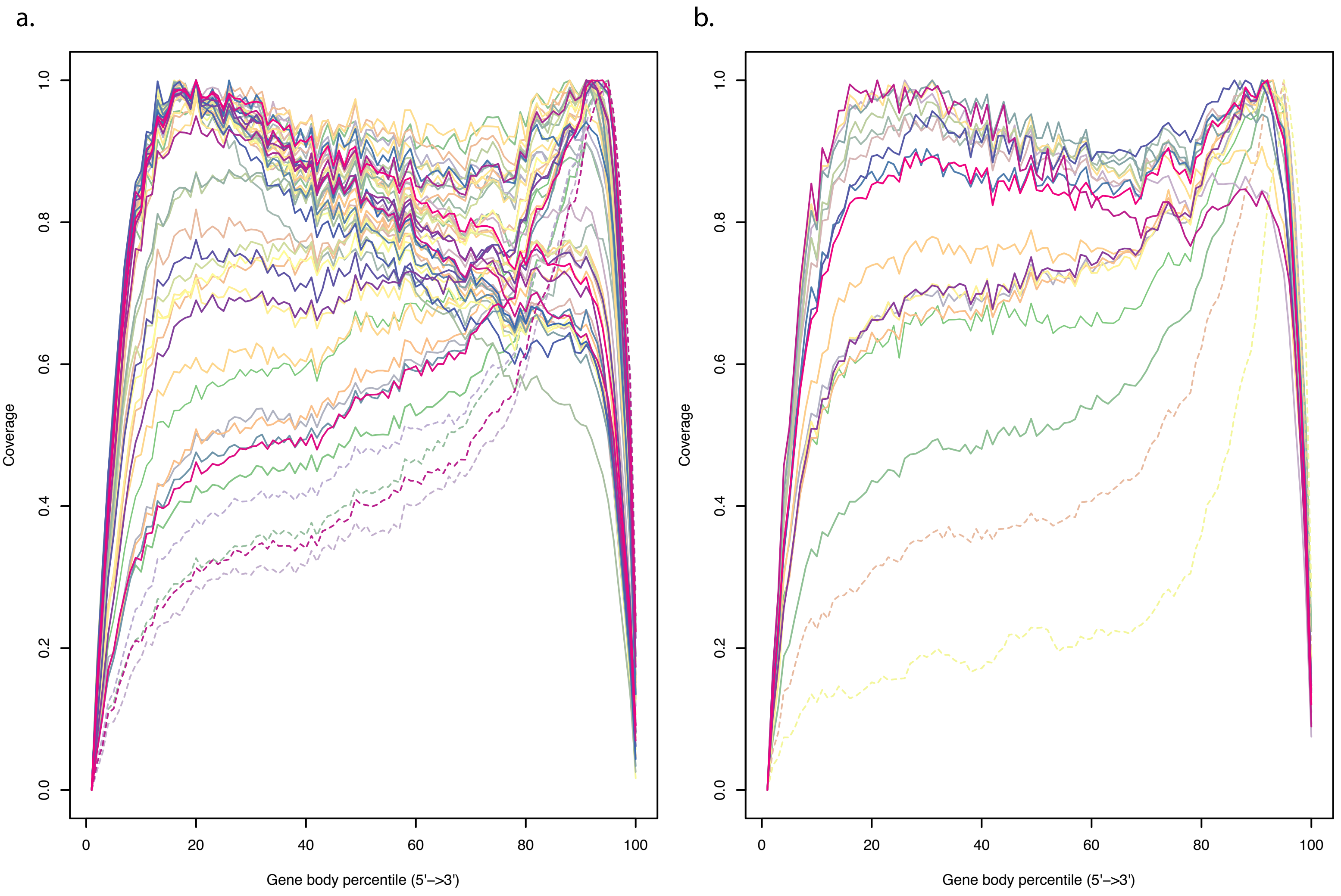
a.
b.
Coverage
0.0
0.2
0.4
0.6
0.8
1.0
0
20
40
60
80
100
Gene body percentile (5'->3')

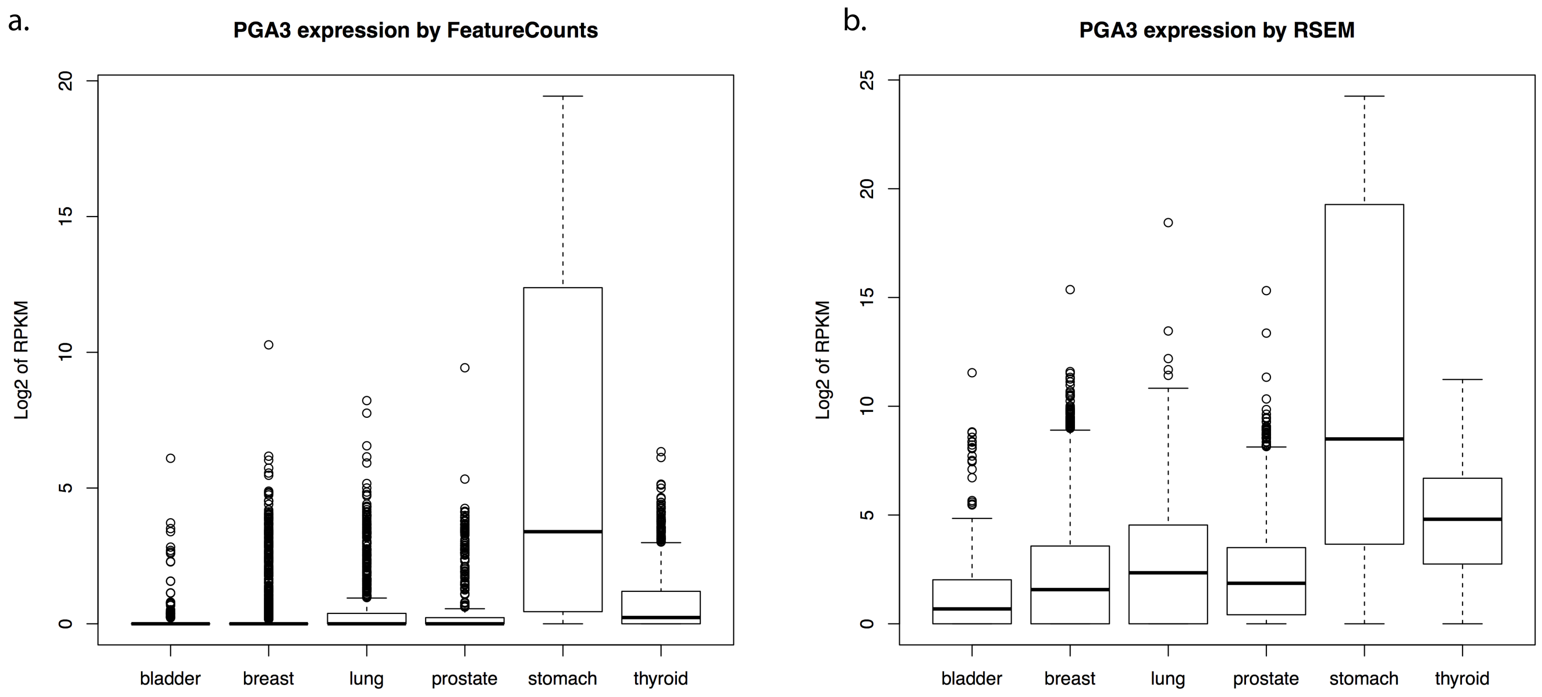
a.
PGA3 expression by FeatureCounts
Log2 of RPKM
0
5
10
15
20
bladder
breast
lung
prostate
stomach
thyroid
b.
PGA3 expression by RSEM
Log2 of RPKM
0
5
10
15
20
25
bladder
breast
lung
prostate
stomach
thyroid

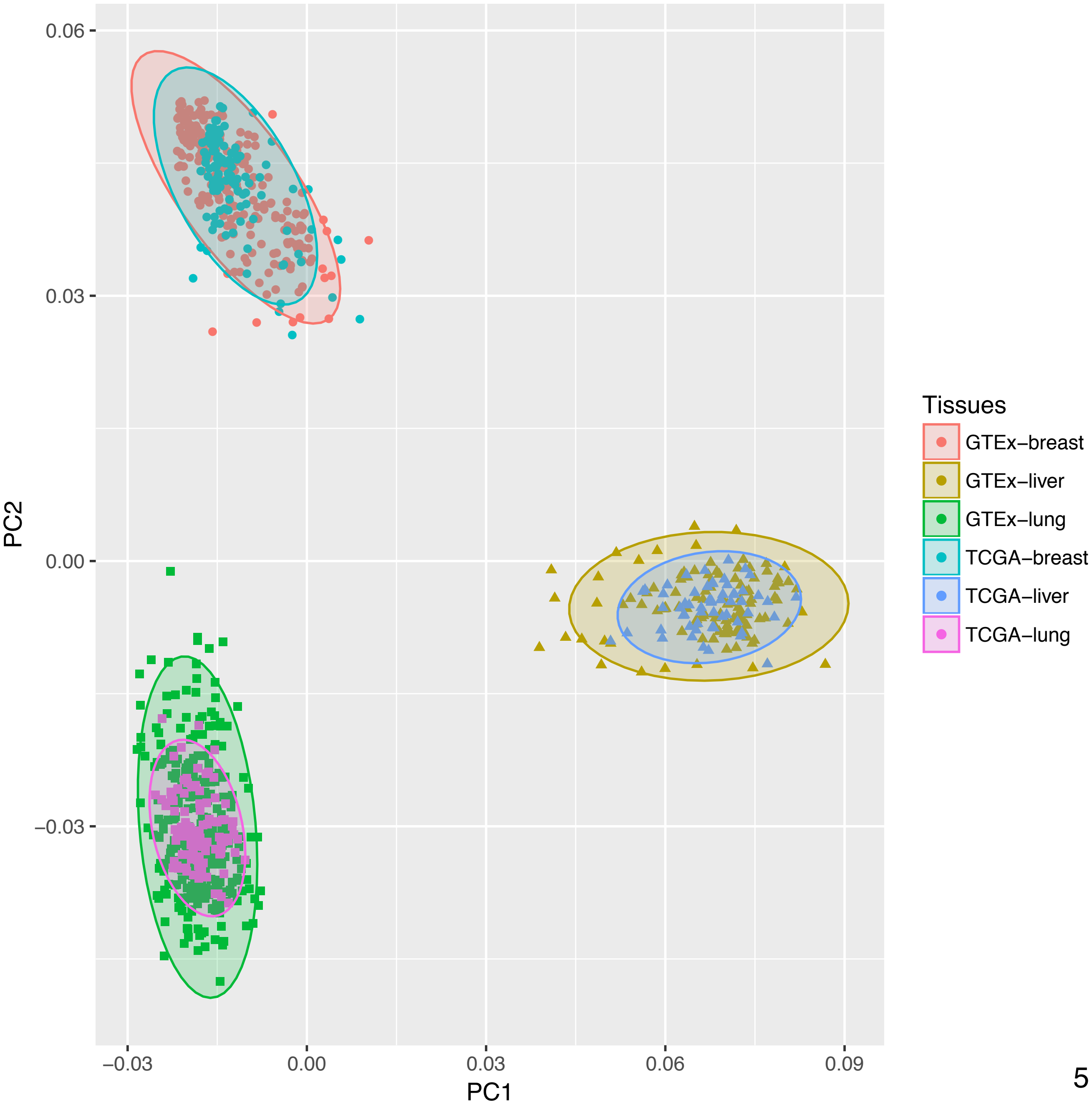
0.06
0.03
0.00
−0.03
PC2
−0.03
0.00
0.03
0.06
0.09
PC1
Tissues
GTEx−breast
GTEx−liver
GTEx−lung
TCGA−breast
TCGA−liver
TCGA−lung

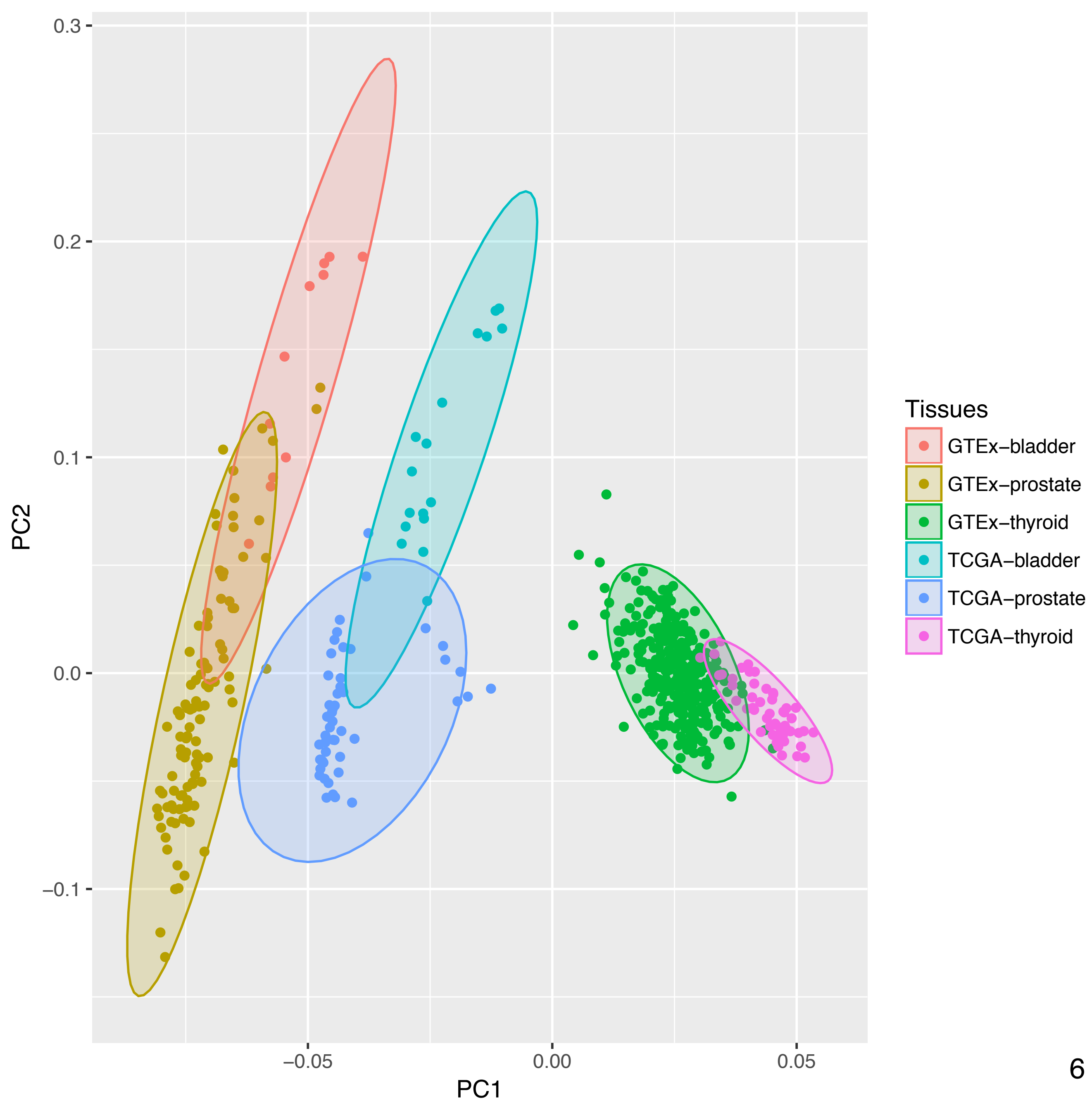
Tissues
GTEx−bladder
GTEx−prostate
GTEx−thyroid
TCGA−bladder
TCGA−prostate
TCGA−thyroid
PC1
PC2
0.3
0.2
0.1
0.0
−0.1
−0.05
0.00
0.05

Table S1. Samples with no or insufficient numbers of normal samples available in TCGA or GTEx.

GTEx tissue / TCGA cancer type	GTEx	TCGA normal	TCGA tumor	Total
adipose / sarc	621	2	259	882
blood / laml	456	0	0	456
none / chol	0	9	36	45
none / dlbc		0	48	48
adrenal gland / acc	159	0	79	238
adrenal gland / pcpg		3	179	182
brain / gbm	1403	0	156	1559
brain / lgg		0	516	516
ovary / ov	108	0	294	402
pancreatic / paad	197	4	178	379
skin / skcm	974	1	103	1078
small intestine / none	104	0	0	104
testis / tgct	203	0	150	353
none / thym		2	120	122
none / meso		0	87	87
none / uvm		0	80	80
**Total**	**4225**	**21**	**2285**	**6531**

Table S2. Parameters of ComBat for: (a) bladder, and (b) lung. TCGA lung cancer has two subtypes: lung adenocarcinoma (LUAD) and lung squamous cell carcinoma (LUSC). LUSC was designated in the same batch as LUAD.

(a) Parameters of ComBat for bladder

	GTEx bladder	TCGA BLCA normal	TCGA BLCA tumor
**Batch**	1	2	2
**Variable of interest**	normal	normal	tumor

(b) Parameters of ComBat for lung

	GTEx lung	TCGA LUAD normal	TCGA LUAD tumor	TCGA LUSC normal	TCGA LUSC tumor
**Batch**	1	2	2	2	2
**Variable of interest**	normal	normal	tumor	normal	tumor
